# Towards a Neuronal Gauge Theory

**DOI:** 10.1371/journal.pbio.1002400

**Published:** 2016-03-08

**Authors:** Biswa Sengupta, Arturo Tozzi, Gerald K. Cooray, Pamela K. Douglas, Karl J. Friston

**Affiliations:** 1 Wellcome Trust Centre for Neuroimaging, University College London, London, United Kingdom; 2 Center for Nonlinear Science, University of North Texas, Denton, Texas, United States of America; 3 Clinical Neurophysiology, Karolinska University Hospital, Stockholm, Sweden; 4 LINT Laboratory, University of California, Los Angeles, Los Angeles California, United States of America

## Abstract

Given the amount of knowledge and data accruing in the neurosciences, is it time to formulate a general principle for neuronal dynamics that holds at evolutionary, developmental, and perceptual timescales? In this paper, we propose that the brain (and other self-organised biological systems) can be characterised via the mathematical apparatus of a gauge theory. The picture that emerges from this approach suggests that any biological system (from a neuron to an organism) can be cast as resolving uncertainty about its external milieu, either by changing its internal states or its relationship to the environment. Using formal arguments, we show that a gauge theory for neuronal dynamics—based on approximate Bayesian inference—has the potential to shed new light on phenomena that have thus far eluded a formal description, such as attention and the link between action and perception.

## Abstract 2

This Essay presents a formalism that not only provides a quantitative framework for modelling neural activity but also shows that neuronal dynamics across scales are described by the same principle.

## Introduction

Recent innovations such as optogenetics and neuroimaging enable us to characterise the relationship between the activity of neurons and their system-level behaviour. These technical innovations call for theories that describe neuronal interactions and reveal the underlying principles. In physics, electromagnetism, the general theory of relativity, and the quantum field theory have been consolidated within the rigorous and holistic framework of a gauge theory [1]. Here, we propose that a gauge-theoretic formalism in neurosciences might not only provide a quantitative framework for modelling neural activity but also show that neuronal dynamics across scales—from single neurons to population activity—are described by the same principle. This paper suggests that if we could formulate a gauge theory for the brain—or cast an existing theory as a gauge theory—then many aspects of neurobiology can be seen as consequences of fundamental invariance properties. This approach could clarify the intimate relationship between apparently distinct phenomena (e.g., action and perception) and, potentially, offer new tools for computational neuroscience and modelling.

This paper is comprised of three sections. The first provides an informal description of gauge theories in physics. The second focuses on the biological features required for a gauge theory for the brain. In the third section, we discuss the three ingredients of a gauge theory: a system equipped with symmetry, some local forces, and one or more gauge fields. To illustrate the approach, we use the principle of variational free energy minimisation as a candidate for a neuronal gauge theory. This principle says that every aspect of the brain, from neuronal activity to synaptic connection strengths, can be described as a process that minimises variational free energy, where variational free energy measures the probability of sensory inputs, under the brain’s model of how those inputs were caused. The variational aspect of variational free energy comes from its derivation from variational calculus, which is at the heart of gauge theories and information geometry, which are the focus of this article.

At its simplest, variational free energy reduces to prediction error or surprise in exactly the same way that estimating the parameters of a statistical model can be reduced to minimising the sum of squared error. We will see later that when physical laws, such as Hamilton's principle of least action, are applied to variational free energy, the resulting behaviour looks very much like statistical inference, lending the brain—and indeed any self-organising system—the look and feel of a system that is trying to estimate or infer the causes of its (sensory) exchanges with the world. In fact, advanced statistical analyses of data minimise variational free energy, where negative free energy approximates Bayesian model evidence. Effectively, minimising variational free energy corresponds to maximising model evidence. This means that if the brain minimises variational free energy it is inherently Bayesian; hence the Bayesian brain hypothesis [2–5].

We provide a glossary of technical terms (S1 Glossary) and series of appendices (S1 Appendix; S1–S5 Text) that offer a technical account of the free energy formalism, gauge theory, and their relationship. These appendices unpack the key conceptual points for technically-minded readers. Although formulating the free energy principle as a gauge theory may have important implications for new inference schemes that can be used to analyse data (i.e., infer the structure and parameters of models of empirical data), we focus on the implications for neurobiology.

## I. Gauge Theories in Physics

A gauge theory is a physical theory that predicts how one or more physical fields interact with matter. Every gauge theory has an associated Lagrangian (i.e., a function that summarises the dynamics of the system in question), which is invariant under a group of local transformations [6]. Consider Newton’s laws of motion in an inertial frame of reference (e.g., a ball in an empty room). These laws are valid at every point in the room. This means that the dynamics and the Lagrangian do not depend on the position of the ball. In this system, the dynamics will be invariant under translations in space. These transformations—that preserve the Lagrangian—are said to be equipped with gauge symmetry. In short, a symmetry is simply an invariance or immunity to changes in the frame of reference [7].

Take a rotating frame of reference (e.g., the Earth) and call it the system. During the rigid rotation of the Earth, the distances between every town remain invariant. As Earth rotates, one can say that we have a gauge transformation that does not change the distance between the cities (S1 Appendix). This means that the system preserves its symmetry (c.f., the laws of Nature), despite the action (or gauge transformations) of global forces (acting on every point of the system). This action corresponds to a global transformation of the system—a transformation that preserves invariances; in this instance, distances. There is, however, another possibility: for example, compare the Earth and moving objects on its surface (e.g., the water in the ocean). When the motion of water is described relative to the Earth, its inertia (that is, its tendency to move in a straight line) distorts its motion. This phenomenon is called the Coriolis effect: rather than flowing directly from areas of high pressure to low pressure—as they would in a nonrotating world—currents tend to flow in a clockwise sense (with respect to the direction of travel) in the Northern hemisphere and counterclockwise in the Southern hemisphere. The Coriolis effect is thus a curvilinear motion in a rotating (noninertial) frame of reference. A gauge transformation that is not constant, i.e., varies with spatial location, is referred to as a local symmetry. One or more local forces acting on just a few parts of the system produce local transformations.

Why is the symmetry preserved, despite the perturbing action of local forces? This is because the local forces are balanced by another force—called the gauge field. Note that when the global symmetry of the system is preserved despite local forces, there is a local deformation, and the symmetry is said to be locally broken, or at best, hidden. In a system equipped with global symmetry, it is thus possible to find a local breaking of symmetry that can be “repaired” by the gauge field. Let us again take the example of the Coriolis forces. For someone who only uses inertial frames of reference, the Coriolis forces do not exist; they are just forces we invent to pretend we are in an inertial system when in fact we are not: they are practical, because they allow us to continue using Newton’s laws. Albert Einstein discovered something similar; the laws of special relativity are not valid when gravitational forces (which are due to energy and momentum) are present. However, by allowing energy and momentum to bend space–time, he could retain special relativity, at least locally, making the gravitational forces disappear. For Einstein, gravitational forces are as unreal as the Coriolis forces; they are forces we have to invent when we choose an inappropriate frame of reference. See S1 Appendix that illustrates these ideas using a simple pendulum example.

In summary, gauge theory is relatively straightforward: one has a Lagrangian that prescribes equations of motion. These equations of motion are generally invariant under changes in a spatial frame of reference (e.g., a straight line is a straight line everywhere on a sheet of paper); however, if we stand back and see that motion (a line in space–time) changes with position (e.g., a straight line on a sheet of paper that has been bent), we can extend the global symmetry to a local symmetry by introducing compensatory fields or forces—the gauge fields. The notion of bending or curvature here is important, because manifolds that shape trajectories in the space of probability distributions are necessarily curved. This is important for systems that minimise variational free energy (S1 Text) and related Bayesian brain theories [8]. In fact, we shall see below that quintessentially cognitive faculties like “attention” can, in principle, be derived using gauge theory.

## II. Gauge Theories for the Nervous System

Gauge theories originate from physics; however, they could be applied to countless fields of biology: cell structure, morphogenesis, and so on. Examples that lend themselves to a gauge-theoretic treatment include recent simulations of embryogenesis [9] and the self-organisation of dendritic spines [10]. Although these examples appear unrelated, both can be formulated in terms of a gradient ascent on variational free energy. In other words, we may be looking at the same fundamental behaviour in different contexts [11]. Here, we focus on the central nervous system (CNS). Can we sketch a gauge theory of brain function? The answer rests on identifying—in biological systems like the brain—the tenets of a gauge theory; namely,

1. A system equipped with a continuous symmetry that can be conserved.
2. The presence of local forces acting on the system (a group of local transformations), where a gauge field renders its Lagrangian invariant under such transformations.

When attempting to establish what aspect of CNS function might be understood in terms of a Lagrangian, the variational free energy looks highly plausible [12]. The basic idea is that any self-organising system, at nonequilibrium steady-state with its environment, will appear to minimise its (variational) free energy, thus resisting a natural tendency to disorder. This formulation reduces the physiology of biological systems to their homeostasis (and allostasis); namely, the maintenance of their states and form, in the face of a constantly changing environment.

If the minimisation of variational free energy is a ubiquitous aspect of biological systems, could it be the Lagrangian of a gauge theory? This (free energy) Lagrangian has proved useful in understanding many aspects of functional brain architectures; for example, its hierarchical organisation and the asymmetries between forward and backward connections in cortical hierarchies [13]. In this setting, the system stands for the brain (with neuronal or internal states), while the environment (with external states) is equipped with continuous forces and produces local sensory perturbations that are countered through action and perception (that are functionals of the gauge field).

In summary, the free energy formalism rests on a statistical separation between the agent (the internal states) and the environment (the external states). Agents suppress free energy (or surprise) by changing sensory input, by acting on external states, or by modifying their internal states through perception [12,14]. In what follows, we try to show that the need to minimise variational free energy (and hence achieve homeostasis) acquires a useful logical mathematical formalism, when framed as a gauge theory.

### The Utility of a Gauge Theory for the CNS

Whenever we posit a normative theory in neuroscience, we appeal to some objective function or Lagrangian—and the opportunity to develop a gauge theory. So what would this gauge theory tell us? Put simply, it would allow us to understand complex and apparently chaotic neuronal dynamics in much simpler yet formal terms. In the same way that we model and understand the gauge fields (e.g., Coriolis forces) driving our weather patterns, it may be possible to understand the driving effects of neuronal afferents on postsynaptic responses as a necessary consequence of much simpler invariances or symmetries. This prospect is particularly prescient if neuronal activity encodes representations or (Bayesian) beliefs, where the Lagrangian (i.e., variational free energy) becomes a function of probability distributions.

Minimising variational free energy describes how a system changes its (internal) states so that they are the most probable under the (external) states of the environment. The system does so by implicitly learning a generative model of its environment. A generative model is simply a model that generates sensory inputs. If the model can reproduce or predict sensory samples from the environment, then it implicitly infers how sensations were generated or caused. Clearly, a good model will make predictions with a very low prediction error (or variational free energy). Put another way, minimising variational free energy ensures the brain becomes a good model of its environment. If we add into this mix the fact that sensory input is actively sampled, then acting to minimise free energy will inevitably make the model’s predictions come true. This is how action and perception are understood in terms of variational free energy minimisation.

Recall that a key ingredient of a gauge theory is a continuous symmetry that can be conserved. So what does gauge symmetry bring to the table? Here, we develop the idea that distinct classes of inference or behaviour are equivalent to pattern formation in the nervous system. For example, the perception of a visual object involves highly organised patterns of neuronal responses throughout the visual cortical hierarchy, reflecting a functional specialisation for colour and motion [15]—right down to the architecture of classical receptive fields [16,17]. Since patterns can be classified into a variety of groups, so can a system’s behaviour. Specifically, the gauge perspective enables us to specify an ontology of patterns or responses that is independent of the generative model. This does not mean that the generative model is irrelevant, but rather a great deal could be learnt by knowing about the symmetries (invariances) of that model. All models with a given symmetry explore the same range of pattern-forming behaviour—without reference to the underlying model [18]. For example, could experience-dependent or Hebbian plasticity [19] be an example of a symmetry that minimises variational free energy [20], and yet the products of experience lead to very different—but equally good—neuronal patterns and architectures. In other words, could we understand phenomena like bistable perception [21] and intersubject variability [22] in terms of a gauge theory.

In the animal kingdom, many aspects of behaviour are similar, yet the neural mechanisms that mediate such behaviours can be very different. For example, bees may forage using different neuronal mechanisms [19] from those used by a rat to explore a maze [23], or those we employ while foraging for information with visual saccades [24]. The difference emerges from the heterogeneity of evolutionary pressures from the ecological niche. A gauge theory that describes behaviour and establishes a mathematical framework to understand the gauge transformations that render behaviour invariant, within and between species, may be invaluable. Particularly because such an approach paves the way for a computational neuroethology, enabling us to study what properties of neuronal dynamics are conserved over species, and what constraints have caused certain neural network properties to diverge over evolution. For example, the minimisation of variational free energy may provide an explanation for foraging that transcends the ecological context: see [25] for an example of epistemic foraging that is formally equivalent to saccadic sampling of the visual field [26].

In the study of dynamical attractors and pattern formation, it generally makes sense to study model-independent generalities as a first step, adding the details later. For example, the Lorenz attractor has found a useful role in modelling a variety of systems; from catalytic reactions [27] to meteorology [28] and immunology [29]. A technical issue here is the distinction between phase and physical space [18]. Any system evolves in a physical space, wherein transformations to a phase-space involve an unspecified change of coordinates. Such coordinate transformations can disconnect variables of the dynamical system and the variables observed in physical space (e.g., the phase reduction techniques used to characterise rhythmic brain activity in terms of fluctuations in the phase of a neuronal oscillation at any given frequency [30] and other dynamical systems ranging from cell biology to cancer research [31]. See also S1 Appendix). The gauge-symmetry perspective alleviates the potentially problematic disconnect between physical and phase spaces: a symmetry property in phase space translates into a symmetry property in the physical space, and vice-versa, because this is the defining property of properly formulated gauge symmetries. A related benefit of understanding the symmetries of a dynamical system is that it enables one to map from one dynamical system to another, allowing us to identify a range of solutions with identical dynamics [32,33].

The second important question posed by framing a gauge theory is "how are symmetries related to gauge fields?" The answer to this question is that gauge fields are objects (compensatory fields) introduced to maintain symmetry and thus also the original equation of motion (S2 Text). In other words, one can imagine them as fields that ensure the Lagrangian is invariant to transformations. In the next section, we use these notions to formulate the variational free energy formalism as a gauge theory for the nervous system.

## III. Sensory Entropy as a Lagrangian

The variational free energy formalism uses the fact that biological systems must resist the second law of thermodynamics (i.e., a tendency to disorder), so that they do not decay to equilibrium. In a similar vein to Maxwell's demon, an organism reduces its entropy through sampling the environment—to actively minimise the self information or surprise of each successive sensory sample (this surprise is upper bounded by free energy). By doing so, it places a bound on the entropy of attributes of the environment in which it is immersed. Variational free energy operationalises this bound by ensuring internal states of the system become a replica (i.e., a generative model) of its immediate environment. This can be regarded as a formulation of the good regulator hypothesis [34], which states that every good regulator of a system must be a model of that system.

We know that a gauge theory would leave the Lagrangian invariant under continuous symmetry transformations. Therefore, a gauge theory of the brain requires the interaction among three ingredients: a system equipped with symmetry, some local forces applied to the system, and one or more gauge fields to compensate for the local perturbations that are introduced. The first ingredient is a system equipped with symmetry: for the purposes of our argument, the system is the nervous system and the Lagrangian is the entropy of sensory samples (which is upper-bounded by variational free energy, averaged over time). The local forces are mediated by the external states of the world (i.e., through sensory stimuli). The gauge fields can then be identified by considering the fact that variational free energy is a scalar quantity based on probability measures. Let us see how.

From differential geometry treatments of probability measures (S3 Text), it is known that the manifold traced out by sufficient statistics of a probability measure is curved in nature [35,36]; more specifically, it has negative curvature and is therefore endowed with a hyperbolic geometry. Barring technical details, it suffices to understand that a functional (e.g., variational free energy) of such probability measures is not flat but induces a curved (Riemannian) landscape. Moving along such a curved manifold requires a measure of distance that corresponds to the distance between two distributions. Again, differential geometry tells us that such a measure of distance is provided by the Fisher information metric (Cencov’s characterisation theorem) that quantifies the amount of information that a random variable carries about an unknown parameter. Technically, the Fisher information represents the curvature of the relative entropy (Kullback-Leibler divergence). Put simply, distances in the curved geometry of sufficient statistics—that define variational free energy—correspond to the relative entropy in going from one point on the free energy manifold to another.

Instead of using classical results from differential geometry [35–39], we will motivate the gauge formulation of variational free energy by asking an empirical question: how does neuronal activity follow the steepest descent direction to attain its free energy minimum? In other words, how does it find the shortest path to the nearest minimum? As the free energy manifold is curved, there are no orthonormal linear coordinates to describe it. This means the distance between two points on the manifold can only be determined with the help of the Fisher information metric that accounts for the curvature. Algebraic derivations (S3 Text) tell us that, in such free energy landscapes, a Euclidean gradient descent is replaced by a Riemann gradient, which simply weights the Euclidean gradient by its asymptotic variance.

In the free energy framework, when the posterior probability is approximated with a Gaussian distribution (the Laplace approximation; S4 Text), perception and action simply become gradient flows driven by precision-weighted prediction errors. Here, prediction errors are simply the difference between sensory input (local perturbations) and predictions of those inputs based upon the systems internal states (that encode probability distributions or Bayesian beliefs about external states that cause sensory input). Mathematically, precision-weighted prediction errors emerge when one computes the Euclidean gradient of the free energy with respect to the sufficient statistics. In a curvilinear space, the precision-weighted prediction errors are replaced by dispersion- and precision-weighted prediction errors. This says something quite fundamental—perception cannot be any more optimal than the asymptotic dispersion (inverse Fisher information) regardless of the generative model. In statistics, this result is known as the Cramér-Rao bound of an estimator. In other words, the well-known bound (upper limit) on the precision of any unbiased estimate of a model parameter in statistics emerges here as a natural consequence of applying information geometry. In the context of the Bayesian brain, this means there is a necessary limit to the certainty with which we can estimate things. We will see next, that attaining this limit translates into attention. See Box 1 for an overview.

**Box 1. How Does Local Gauge Invariance Explain Neuronal Inference?**

This essay considers the principle of free energy minimization as a candidate gauge theory that prescribes neuronal dynamics in terms of a Lagrangian. Here, the Lagrangian is the variational free energy, which is a functional (a function of a function) of a probability distribution encoded by neuronal activity. This probabilistic encoding means that neuronal activity can be described by a path or trajectory on a manifold in the space of sufficient statistics (variables that are sufficient to describe a probability distribution). In other words, if one interprets the brain as making inferences, the underlying beliefs must be induced by biophysical representations that play the role of sufficient statistics. This is important because it takes us into the realm of differential geometry (S2 Text), where the metric space—on which the geometry is defined—is constituted by sufficient statistics (like the mean and variance of a Gaussian distribution). Crucially, the gauge theoretic perspective provides a rigorous way of measuring distance on a manifold, such that the neuronal dynamics transporting one distribution of neuronal activity to another is given by the shortest path. Such a free energy manifold is curvilinear, and finding the shortest path is a nontrivial problem—a problem that living organisms appear to have solved. It is at this point that the utility of a gauge theoretic approach appears; suggesting particular solutions to the problem of finding the shortest path on curved manifolds. The nature of the solution prescribes a normative theory for self-organised neuronal dynamics. In other words, solving the fundamental problem of minimising free energy—in terms of its path integrals—may illuminate not only how the brain works but may provide efficient schemes in statistics and machine learning.

Variational or Monte Carlo formulations of the Bayesian brain require the brain to invert a generative model of the latent (unknown or hidden) causes of sensations (S3 Text). The implicit normative theory means that neuronal activity (and connectivity) maximises Bayesian model evidence or minimises variational free energy (the Lagrangian)—effectively fitting a generative model to sensory samples. This entails an encoding of beliefs (probability distributions) about the latent causes, in terms of biophysical variables whose trajectories trace out a manifold. In (deterministic) variational schemes, the coordinates on this manifold are the sufficient statistics (like the mean and covariance) of the distribution or belief, while for a (stochastic) Monte Carlo formulation, the coordinates are the latent causes themselves (S3 Text). The inevitable habitat of these sufficient statistics (e.g., neuronal activity) is a curved manifold (see S1 and S2 Figs).

This curvature (and associated information geometry) may have profound implications for neuronal dynamics and plasticity. It may be the case that neuronal dynamics—or motion in some vast neuronal frame of reference—is as simple as the pendulum (S1 Appendix). However, because the Lagrangian is a function of beliefs (probabilities), the manifold that contains this motion is necessarily curved. This means neuronal dynamics, in a local frame of reference, will (appear to) be subject to forces and drives (i.e., Levi-Civita connections). For example, the motion of synaptic connection strengths (sufficient statistics of the parameters of generative models) depends upon the motion of neural activity (sufficient statistics of beliefs about latent causes), leading to experience-dependent plasticity. A more interesting manifestation (highlighted in the main text) may be attention that couples the motion of different neuronal states in a way that depends explicitly on the curvature of the manifold (as measured by things like Fisher information). In brief, a properly formulated gauge theory should, in principle, provide the exact form of neuronal dynamics and plasticity. These forms may reveal the underlying simplicity of many phenomena that we are already familiar with, such as event-related brain responses, associative plasticity, attentional gating, adaptive learning rates, and so on.

Notice that the definition of a system immersed in its environment can be extended hierarchically, wherein the gauge theory can be applied at a variety of nested levels. At every step, as the Lagrangian is disturbed (e.g., through changes in forward or bottom-up sensory input), the precision-weighted compensatory forces change to keep the Lagrangian invariant via (backward or top-down) messages. In the setting of predictive coding formulations of variational free energy minimisation, the bottom-up or forward messages are assumed to convey prediction error from a lower hierarchical level to a higher level, while the backward messages comprise predictions of sufficient statistics in the level below. These predictions are produced to explain away prediction errors in the lower level. From the perspective of a gauge theory, one can think of the local forces as prediction errors that increase variational free energy, thereby activating the gauge fields to explain away local forces [40]. In this geometrical interpretation, perception and action are educed to form cogent predictions, whereby minimization of prediction errors is an inevitable consequence of the nervous system minimising its Lagrangian. Crucially, the cognitive homologue of precision-weighting is attention, which suggests gauge fields are intimately related to (exogenous) attention. In other words, attention is a force that manifests from the curvature of information geometry, in exactly the same way that gravity is manifest when the space–time continuum is curved by massive bodies. In summary, gauge theoretic arguments suggest that attention (and its neurophysiological underpinnings) constitutes a necessary weighting of prediction errors (or sensory evidence) that arises because the manifolds traced out by the path of least free energy (or least surprise) are inherently curved.

## Conclusions

The importance of the reciprocal interactions among different scales and between the nervous system and the world has been repeatedly emphasised here and elsewhere. A quantitative formulation of this holistic aspect of information processing in the brain is clearly needed and has been framed in terms of variational free energy minimisation or, more simply, as a suppression of prediction errors encountered by an agent that is actively sampling its environment [12,14]. In this essay, we have reformulated the resolution of prediction error (or surprise) as a gauge theory for the nervous system. Such geometrical formulations endow variational inference with symmetry breaking, enabling invariance with respect to the generative model; irrespective of the model’s parameterization [41,42].

**Fig 1:**
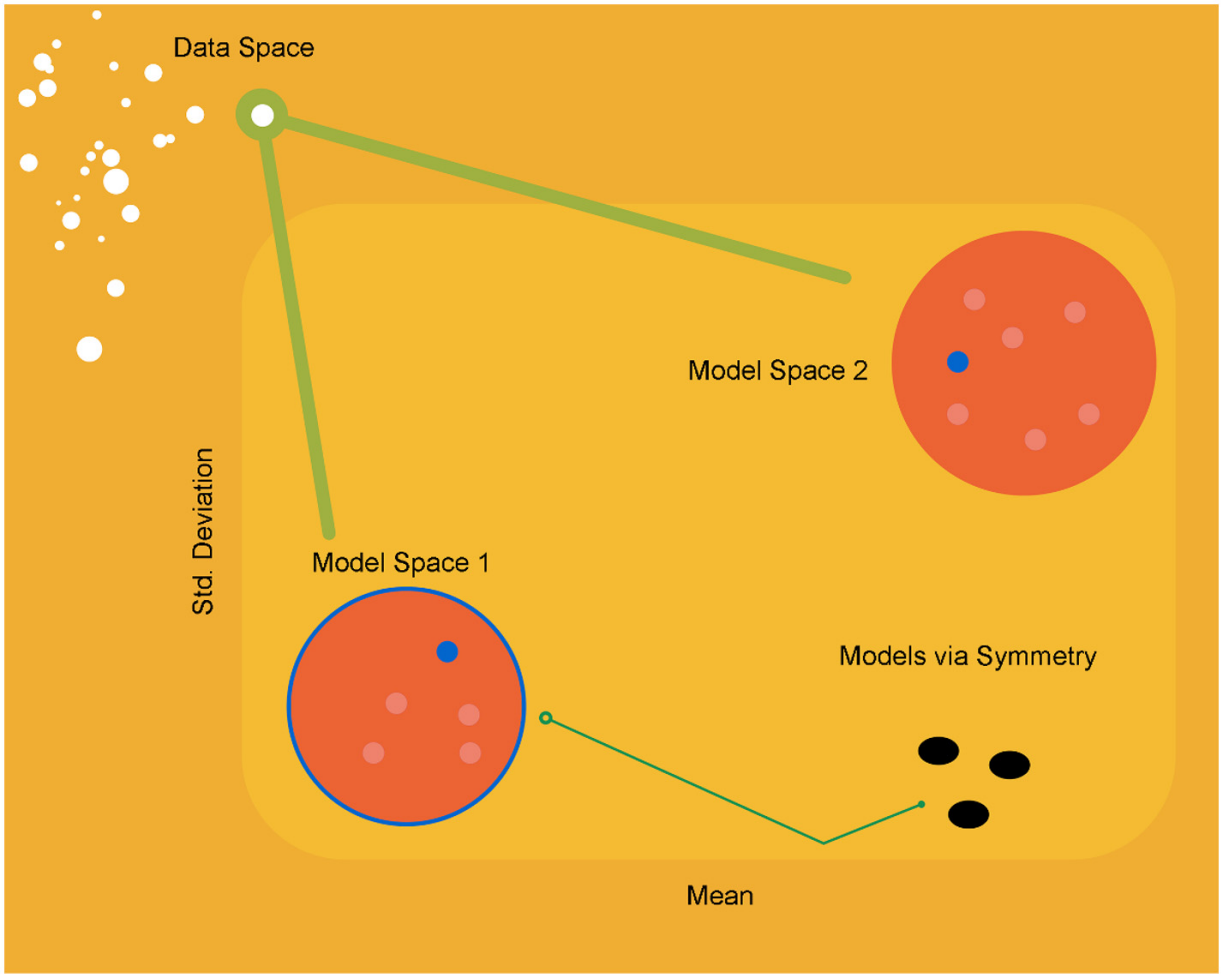
Invariance of inference—a proposal. A univariate normal model space (of the equivalent data space) whose coordinates are sufficient statistics. The orange circles represent two model spaces, where minimization of variational free energy leads to the appropriate model (blue circles in each case). It might be that one parameterisation (blue outer circle for model space 1) is more suitable than another. Notice that using the Riemann gradient instead of the Euclidean gradient automatically guarantees gauge-invariance by breaking the symmetry within a model. But what about between-model symmetries? After the optimal model has been selected, symmetry transformation could enable one to derive a range of models (black circles) that describe the data equally well (in terms of log model evidence or negative free energy). These can be obtained by using the intrinsic geometry of the gauge field (the Levi-Civita connection) and the symmetries afforded by the variational free energy.

The free energy formulation enables us (or our brains) to compare a variety of hypotheses about the environment given our sensory samples. So what does a gauge formulation add? After scoring a variety of models according to their variational free energy, one selects the best generative model that explains the data at hand. If symmetry transformations can be evaluated for the best performing model, afforded by the symmetry group, we can possibly go a step further and ask what other models could explain the data with the same model evidence? Such symmetry transformations therefore furnish us with a family of models that explain the data with equal probability [43–45]: see Fig 1 for a schematic illustration of this idea. The challenge of such an approach would be to deduce the symmetry groups of the probability distributions encoded by neuronal activity (S5 Text). If this was possible, one could identify the class of models explaining data using a single inference methodology. From the perspective of biological self-organisation, this means that there are potentially many different models that do the same job of predicting sensory data. It is tempting to associate the equivalence classes afforded by symmetry groups above with the biological species—all doing an equally good job of modelling their (local) environments.

## Supporting Information

S1 Appendix Global and local gauge invariance. (DOCX)

S1 Fig Conjugate gradient-descent algorithm on manifolds. (A) New parameters (*θ*) are selected by performing gradient descent on orthogonal subspaces with gradient *G* and the descent direction *H*. (B) On a Riemannian manifold, minimization along lines (as in a Euclidean subspace described in A) is replaced by minimization along geodesics. This creates a problem, in that *H*_*i*_ and *H*_*i−1*_ are in two different tangent spaces and thus cannot be added together. (C) Vector addition as in Eqn. 12 (in S3.2) is undefined on a Riemannian manifold. Addition is replaced by exponential mapping followed with parallel transport described using a covariant gauge field (Levi-Civita connection; see text).
(TIFF)

S2 FigParallel transport on Riemannian manifolds.(A) The Riemann exponential map is used to map a vector field *H* from TM→M whilst a logarithmic map is used to map the vector field from M→TM. (B) Graphical illustration of parallel transporting a vector field *H* using a Schild’s ladder (see text for details).(TIFF)

S1 Glossary (DOCX)

S1 Text Variational free energy. (DOCX)

S2 Text A tutorial on differential geometry. (DOCX)

S3 Text Dynamics on manifolds. (DOCX)

S4 Text The geometry of a univariate normal distribution. (DOCX)

S5 Text Symmetry-aware algorithms. (DOCX)
