# Supplementary material for: Towards a Neuronal Gauge Theory
Source: PLoS Biol. 2016 Mar 8;14(3):e1002400. doi: 10.1371/journal.pbio.1002400 (PMC4783098; doi:10.1371/journal.pbio.1002400)
Supplement: S1 Appendix — (DOCX) [file pbio.1002400.s001.docx]

**S1 Appendix. Global and local gauge invariance**

Consider a simple pendulum that oscillates with the same frequency $\omega$ in *x* and *y* coordinates. It is usually convenient to use polar coordinates, where $z=x+iy$, such that the motion can be described by a (second order) differential equation $\partial_{tt}z+\omega^{2}z=0$ and the direction or angle satisfies $\tan\theta=y/x$. In terms of the motion, the absolute coordinates (the *x* and *y* axes) are simply irrelevant; for example, we can rotate the axes by $\alpha$ to represent the motion in new coordinates $x'$ and $y'$. The differential equation remains the same (although we need to replace $\theta$ by $\theta-\alpha$). This amounts to multiplying the differential equation by $e^{-i\alpha}$ and redefining $z'=ze^{-i\alpha}$. This means that although we have rotated the frame of reference, the equation of motion is unchanged. This is **global gauge invariance**. It is global because the entire frame of reference was rotated by the same amount, irrespective of where or when we describe the motion.

Now imagine we rotate the *x* and *y* co-ordinates by an angle that depends on time: i.e., the rotation $\alpha$ now becomes $\alpha(t)$. With this local transformation (local with respect to a particular time), multiplying by $e^{-i\alpha}$ does not ensure the invariance of the differential equation. How could we then retain the laws of motion? Remarkably, we can do so by replacing the time derivatives by $\partial_{t}-iA(t)$. The field $A(t)$ is the compensatory **gauge field** that ensures the equation of motion holds, locally. This is **local gauge invariance**. This amounts to rotating our coordinate system with an angular velocity $\partial_{t}\alpha(t)$ creating a fictitious force (like a Coriolis force). In short, the gauge field $A(t)$ induces compensatory yet fictitious forces, necessary to retain invariance under a local gauge transformation. From a local perspective, the pendulum will appear to move erratically in response to the gauge field; while, from a global perspective, nothing has changed.
