# Supplementary figures and images for: Towards a Neuronal Gauge Theory
Source: PLoS Biol. 2016 Mar 8;14(3):e1002400. doi: 10.1371/journal.pbio.1002400 (PMC4783098; doi:10.1371/journal.pbio.1002400)

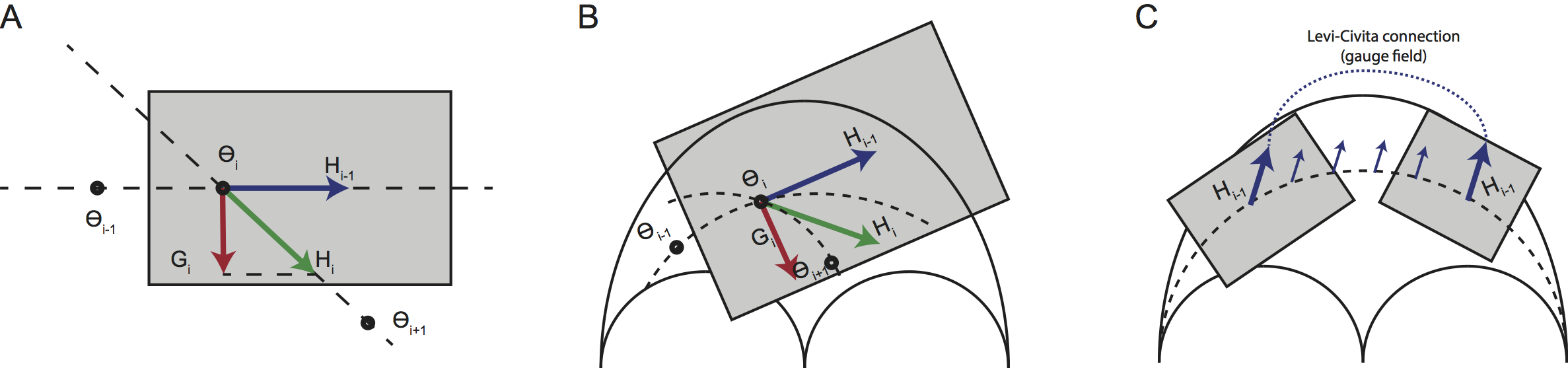

Supplement: S1 Fig — (A) New parameters (θ) are selected by performing gradient descent on orthogonal subspaces with gradient G and the descent direction H. (B) On a Riemannian manifold, minimization along lines (as in a Euclidean subspace described in A) is replaced by minimization along geodesics. This creates a problem, in that H i and H i−1 are in two different tangent spaces and thus cannot be added together. (C) Vector addition as in Eqn. 12 (in S3.2) is undefined on a Riemannian manifold. Addition is replaced by exponential mapping followed with parallel transport described using a covariant gauge field (Levi-Civita connection; see text). (TIFF) [file pbio.1002400.s002.tiff]

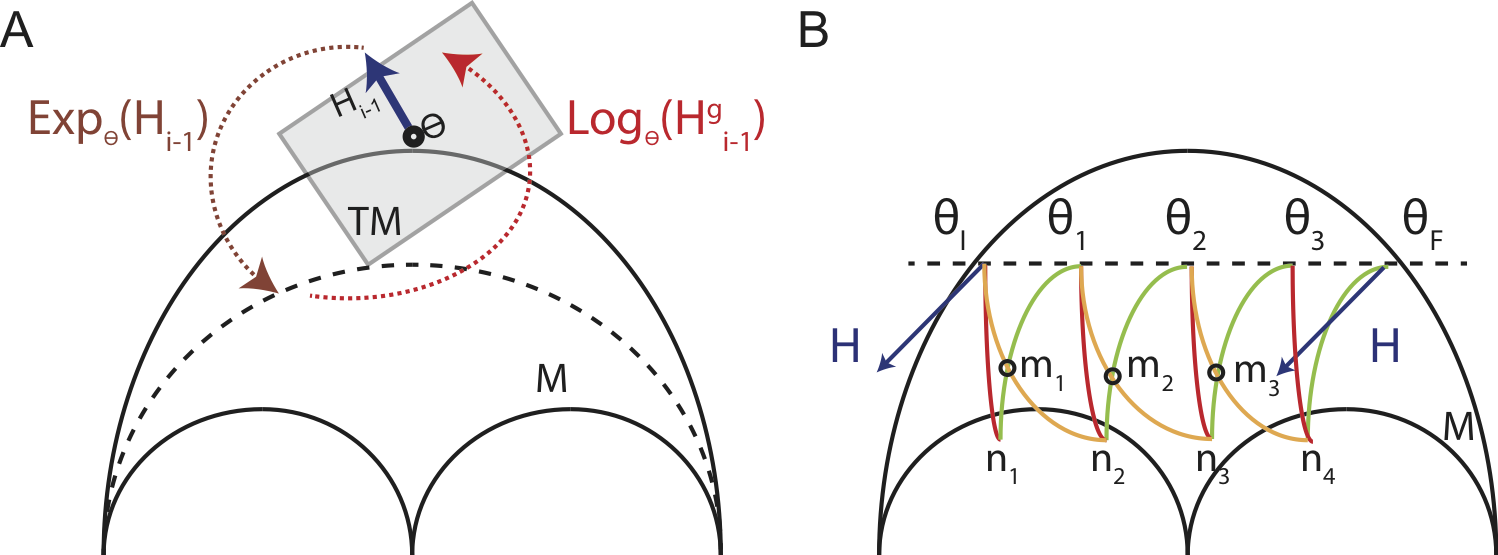

Supplement: S2 Fig — (A) The Riemann exponential map is used to map a vector field H from TM→M whilst a logarithmic map is used to map the vector field from M→TM. (B) Graphical illustration of parallel transporting a vector field H using a Schild’s ladder (see text for details). (TIFF) [file pbio.1002400.s003.tiff]
