# Supplementary material for: Towards a Neuronal Gauge Theory
Source: PLoS Biol. 2016 Mar 8;14(3):e1002400. doi: 10.1371/journal.pbio.1002400 (PMC4783098; doi:10.1371/journal.pbio.1002400)
Supplement: S1 Glossary — (DOCX) [file pbio.1002400.s004.docx]

**S1 Glossary**

- **Lagrangian mechanics**: It is an alternative way to look at Newton’s laws of motion. Put simply, it describes the motion of an object by first formulating a function (the Lagrangian) that is the difference between the object’s kinetic and potential energy, and then finding the minimum of this function over time (this is called action). Newton’s laws of motion fall out as a result of this minimization– this is known as the principle of least action.
- **Invariance and equivariance**: A function $f$ is invariant under a transformation $g$ when $f(g(x))=f(x)$. The function is equivariant when$f(g(x))=\left|\partial g/\partial x\right|f(x)$. For example, the distances between points in a picture do not change when the picture is rotated (invariance), while the angles between the points do not change when the picture is enlarged (equivariance).
- **Gauge theory**: It is any field theory (i.e., a specification of how a field of quantities changes with time or other variables), where the field’s Lagrangian is invariant under a continuous group of local transformations.
- **Gauge fields**: These can be viewed as fictitious forces (mathematically, an Ehresmann connection) that are introduced to compensate for a poorly chosen coordinate system; such that there exists no gauge (or coordinate) transformation that can make the gauge field vanish.
- **Group theory**: The study of algebraic structures known as groups. Put simply (everyday) objects such as fields or vector spaces are groups. The distinction between different sorts of groups rests on the types of operations and axioms that can be applied.
- **Riemann geometry**: It is a branch of differential geometry that studies smooth manifolds (i.e. surfaces) with a measure of distance instantiated as an inner product (metric).
- **Information geometry**: Using insights from Riemann geometry, information geometry studies the properties of statistical manifolds such as those defined by probability distributions.
- **Symmetry**: Symmetry is any property of a system that is invariant under certain transformations. For example, an equilateral triangle remains the same, when rotated by 60°.
- **Fisher information**: It quantifies the amount of information that an observable variable carries about an unknown (random) variable causing the observation.
- **Kullback-Leibler divergence**: A non-commutative measure (and non-negative) of the difference between two probability distributions.
- **Free energy**: An information theoretic measure that bounds or limits (by being greater than) the surprise (or self information) on sampling some data, under a model of how those data are generated.
- **Entropy**: The average surprise (self information) of outcomes sampled from a probability distribution. A distribution with low entropy means that, on average, the outcome is predictable. Entropy is therefore a measure of uncertainty.
- **Sufficient statistics**: These are quantities that are sufficient to parameterize a probability density (for example, the mean and covariance of a Gaussian distribution).
- **Generative model**: A probabilistic model (joint distribution) of the dependencies between causes and effects (data), from which samples can be generated. It is usually specified in terms of the likelihood of data, given their causes (parameters of a model) and priors over those causes.
- **Prior**: The probability distribution of the causes of data that encodes beliefs about those causes before observing the data.
- **Bayesian brain hypothesis**: The idea that the brain uses internal probabilistic (generative) models to update posterior beliefs, using sensory information, in a (approximately) Bayes-optimal fashion.
- **Laplace approximation**: A saddle-point approximation of the integral of a function that uses a second-order Taylor expansion. When the function is a probability density, the implicit assumption is that the density is approximately Gaussian.
