# Supplementary material for: Towards a Neuronal Gauge Theory
Source: PLoS Biol. 2016 Mar 8;14(3):e1002400. doi: 10.1371/journal.pbio.1002400 (PMC4783098; doi:10.1371/journal.pbio.1002400)
Supplement: S1 Text — (DOCX) [file pbio.1002400.s005.docx]

**S1 Text. Variational free energy**

In these appendices (supporting information), we provide a brief overview of the ingredients required to establish a gauge-theoretic formulation of variational free energy minimisation. We start by describing the basics of variational free energy followed by a primer on differential (Riemann) geometry, and ending with the propagation of probability measures on a curved manifold. Throughout, Einstein summation over repeated indices is assumed.

The variational free energy formalism assumes that an agent minimizes the entropy of its sensory states$s\in S$. Only through its sensory receptors can a biological system access the states of its environment; in other words, sensory states form a veil (technically, a Markov blanket) between the system’s internal states $\theta\in\Theta$ and its environment (external states)$\psi\in\Psi$. By bounding the entropy of its sensory states, the system confines the entropy of its environment. Under the assumption of ergodicity, this entropy is the long-term average of surprise. Crucially, the system cannot calculate this quantity directly because it has to marginalize over the external states that cause sensory input. The objective then becomes to obtain a lower bound on the marginal likelihood $\ln p(s)$ by approximating it using a parametric probability distribution (for example, a Gaussian distribution) $q(\psi)$ over the (unknown) external states that are hidden behind the Markov blanket (i.e., hidden causes of sensations). In short, the Lagrangian is minimised by bounding the surprise using the variational free energy of the distribution $q(\psi|\theta)$ where $\theta$ are the sufficient statistics or parameters of the variational distribution. In the case of a Gaussian distribution the sufficient statistics are simply the mean and the co-variances $\{\mu,\sum\}\subset\theta$.

The variational distribution that minimises free energy can be expressed in terms of an Euler-Lagrange action , implying that the gradient descent on the variational free-energy manifold leads us to the most optimal representation of the external states. A numerical scheme to solve such a variational problem is generalised (Bayesian) filtering [[1](#_ENREF_1)]. The Bayesian perspective follows because our Lagrangian is also known as (the negative logarithm of) Bayesian model evidence. In other words, minimising free energy is equivalent to maximising model evidence.

1. Friston KJ, Trujillo-Barreto N, Daunizeau J (2008) DEM: A variational treatment of dynamic systems. Neuroimage 41: 849-885.
