# Supplementary material for: Towards a Neuronal Gauge Theory
Source: PLoS Biol. 2016 Mar 8;14(3):e1002400. doi: 10.1371/journal.pbio.1002400 (PMC4783098; doi:10.1371/journal.pbio.1002400)
Supplement: S2 Text — (DOCX) [file pbio.1002400.s006.docx]

**S2 Text. A tutorial on differential geometry**

In this section, we present a brief introduction to key concepts in differential (Riemann) geometry that are necessary to understand information geometry. Biological systems are immersed in a 4-dimensional world where 3-spatial and 1-time co-ordinates form the frame of reference. Fibre bundles are constructs from differential geometry that offers a general formulation of such frames of reference. Intuitively, a 2-dimensional sheet is sampled by gluing together a collection of identical 1-dimensional lines, stacked, say, on the x-axis. The lines are then called fibres ($F$) and the subspace that glues them together is called the base ($B$) – taken together, the lines form a fibre bundle ($\bar{F}$). Replacing the 2-dimensional sheet by a cylinder simply alters the base; wherein the fibres are attached to a circle and the co-ordinates of a line range between plus and minus infinity, while the co-ordinates on a circle take values between 0 and 2$\pi$. In such a case – where it is easy to find a global co-ordinate system – we call the corresponding bundle *trivial*. A non-trivial bundle would be a Möbius strip, where it is not possible to instantiate a global co-ordinate system. Such scenarios occur more often than not. In these cases, it is possible to use a web of local co-ordinate systems. The only requirement then becomes establishing some rules that describe how a local co-ordinate changes between adjoining patches. A connection field – also known as the gauge field – fulfils this requirement and reconciles the apparent disagreement between the coordinate systems of the fibres glued to nearby points of the base.

The gauge field is therefore a vector field comprised of independent coordinates in the base. For the comparison of two co-ordinate systems – of two local points – it suffices to construct a rule for frame adjustments along the independent coordinates of the base. The difference can be accounted by a series of transitions along the independent directions (say $b$). Mathematically, one calculates the scalar product of the difference between the points of the base $db$ and the gauge field$A$

$\Delta F(b)=db\cdot AF\equiv\sum_{i=1}^{\dim B} A^{i}F(b).db^{i}$.

Here the gauge field $A$ is a transformation between the coordinate systems of $F(b)$ and $F(b+db)$. The route between$F(b)$ and $F(b+db)$ defines a curve; along with the gauge field, these define a parallel transport along the curve. When we compare two distant elements of a fibre using a connection field – and do not find a difference – we can say that the second element is a result of parallel transport of the first element along the curve. Let us now formalize the concepts that we have described so far:

**Definition 1.** A **smooth fibre bundle** is a composite object made up of – (a) a smooth manifold $\bar{F}$that is called the total (bundle) space, (b) a smooth manifold $B$ called the base space, (c) a smooth mapping $w:\bar{F}\to B$ called the projection whose Jacobian is required to have maximal rank at every point, (d) a smooth manifold $F$called the fibre and (e) a group $G$ of smooth transformations of the fibre $F$called the structure group of the fibre bundle. **Tangent** and **co-tangent** bundles are special cases of a fibre bundle. The Lagrangian, summarizing the dynamics is the natural energy function on the tangent bundle whilst the Hamiltonian is the natural energy function on the co-tangent bundle. A **jet bundle** generalizes both the tangent and the cotangent bundle.

**Definition 2.** A fibre bundle is characterised as ***trivial*** if we can introduce a global co-ordinate system so that any point can be identified using a pair of co-ordinates $(x,f)$ where $f$ is a set of co-ordinates on the fibre and $x$ is a set of co-ordinates on the base. Similarly, a ***principal fibre bundle*** is a special fibre bundle, whose fibre is the group $G$.

**Definition 3.** For a curve $\gamma$ on the base, a **connection** is a map of the fibre $F_{\gamma(a)}$ at point $\gamma(a)$ to fibre $F_{\gamma(b)}$ at point $\gamma(b)$ that satisfy the following requirements – (a) depends continuously on $\gamma(t)$, (b) is independent of the parameterization of the path – parallel transport is defined by the path rather than the function $\gamma(\cdot)$, (c) is the identity map if $\gamma(t)$ is constant and (d) parallel transport along two consecutive curves is equivalent to transport along the combined curve; a corollary of which states that transport in opposite direction generates the inverse parallel transport. A connection is called a **G-connection** if the map is an element of the structure group for any curve in the base. In summary, results of parallel transport with the same endpoint but different paths can be different. The **curvature** of the fibre bundle measures this difference.

For our purposes, we will confine ourselves to a Riemannian manifold – a manifold that is analytic and where each tangent space is equipped with an inner product, varying smoothly from point to point. This inner product takes the form of a metric on the tangent bundle that approximates the manifold locally. This enables us to define various notions such as length, angles, volumes, etc.

**Definition 4.** A **Riemannian metric** on a manifold is a covariant 2-tensor $g$ which associates each point on the manifold with an inner product $g=\left\langle-,-\right\rangle_{x}$ on the tangent space . The metric is not only bilinear but also symmetric and positive definite and therefore defines a Euclidean distance on the tangent space. In terms of local co-ordinates the metric is given by a matrix $g_{ij}=\left\langle X_{i},X_{j}\right\rangle_{x}$ where $X_{i}$ and $X_{j}$ are tangent vectors to at $x$ and varies smoothly with$x$. A **geodesic** curve is a local minimiser of arc-length computed with a Riemannian metric.

**Definition 5.** In Riemann geometry, the **Levi-Civita connection** is the torsion or curvature free connection of the tangent bundle that preserves the Riemannian metric. More specifically, it is a unique affine connection such that it is

- compatible with metric i.e., $X.g(Y,Z)=g(\nabla_{X}Y,Z)+g(Y,\nabla_{X}Z)$
- has symmetry i.e., $\nabla_{X}Y-\nabla_{Y}X=[X,Y]$ where $[X,Y]$ is the Lie bracket

The compatibility condition can be expressed in terms of covariant derivative i.e., if $X(t)=X(\gamma(t))$ and $Y(t)=Y(\gamma(t))$ are two vector fields along the curve $\gamma$ and $D/dt$ is the co-variant derivative along $\gamma$ then,

$$\frac{d}{dt}g\left(X(t),Y(t)\right)=g\left(\frac{DX(t)}{dt},Y(t)\right)+g\left(X(t),\frac{DY(t)}{dt}\right)$$

**Definition 6.** In a manifold that has been parameterised and the curve $\gamma(t)$ is represented as $(x^{1}(t),\ldots,x^{M}(t))$, the co-variant derivative of a vector field $v$ can be written as,

$$\frac{Dv}{dt}=\sum_{m} \left\{\frac{dv^{m}}{dt}+\sum_{i,j} \Gamma_{ij}^{m}v^{j}\frac{dx^{i}}{dt}\right\}\frac{\partial}{\partial x_{m}}$$

Here, the coefficients of the connection $\Gamma_{ij}^{m}$ are known as the **Christoffel symbols**. Simply, the parallel transport along the curve becomes a first order linear system,

$$\frac{dv^{m}}{dt}+\sum_{i,j} \Gamma_{ij}^{m}v^{j}\frac{dx^{i}}{dt}=0,\text{ m=1,}\ldots\text{,M}$$

In such a case the Christoffel symbols are given by,

$$\Gamma_{ij}^{m}=\frac{1}{2}\sum_{l} \left\{\frac{\partial}{\partial x_{i}}g_{jm}+\frac{\partial}{\partial x_{j}}g_{mi}-\frac{\partial}{\partial x_{m}}g_{ij}\right\}g^{ml}$$

$g^{ml}$ represents the metric inverse. The geodesic equation can now be written as a system of a second order system,

$$\frac{d^{2}x^{m}}{dt^{2}}+\sum_{i,j} \Gamma_{ij}^{m}\frac{dx^{i}}{dt}\frac{dx^{j}}{dt}=0,\text{ m=1,}\ldots\text{,M}$$

This concludes our brief introduction to differential geometry.
