# Supplementary material for: Towards a Neuronal Gauge Theory
Source: PLoS Biol. 2016 Mar 8;14(3):e1002400. doi: 10.1371/journal.pbio.1002400 (PMC4783098; doi:10.1371/journal.pbio.1002400)
Supplement: S3 Text — (DOCX) [file pbio.1002400.s007.docx]

**S3 Text. Dynamics on manifolds**

We will now use the concepts described in Section S2 to describe the geometry of the variational free-energy landscape (Section S1). We begin by formulating the evolution of parameters using Langevin equations (a stochastic formulation) before moving on to describe the evolution of the approximate probability distribution, as used in the variational free-energy framework (a deterministic formulation).

**S3.1 Stochastic approach** – parameters of the generative model form the co-ordinate basis

The evolution of parameters $\theta$ of a generative model can be characterised with a Langevin equation, whose ensemble density minimises variational free energy [[1](#_ENREF_1)],

where we define the quantity and $\mathbf{b}$ denotes Brownian motion. It is important to realize that this Langevin equation describes the stochastic flow of parameters – and not of the sufficient statistics of the distribution that describes the parameters (see section S3.2). In theory such a stochastic differential equation (SDE) should converge to a unique and invariant ergodic density, which will approximate the posterior density of parameters given sensory data. Discretisation using numerical methods often lead to non-convergence or convergence to a ‘wrong’ ergodic density. An empirical solution amounts to probabilistically accepting solutions at each time step depending on the relative densities of the solution at the new and the old time-steps (e.g., Markov chain Monte Carlo procedures).

By virtue of the Nash embedding theorem, our aim here is to define the above (Langevin) diffusion process on a manifold that is embedded on a higher dimensional Euclidean space. If we can do this, the evolution of the probability measure on the manifold simply becomes an Euler-Lagrange flow.

Before we start our derivation, let us generalise the Laplace operator to Riemannian manifolds. This linear operator is known as the Laplace-Beltrami operator, composed as the divergence of the covariant derivative [[2](#_ENREF_2)]. Assume that is an oriented Riemannian manifold, then the volume form on indexed by the coordinate system $\theta^{i}$ is

$$\text{vol}_{\text{n}}=\sqrt{\left|g\right|}d\theta^{1}\wedge d\theta^{2}\ldots d\theta^{n}$$

where $d\theta^{i}$ are the 1-forms forming a dual basis, $g$ is the metric tensor and is the familiar wedge product. The divergence of a vector field on a manifold is then a scalar function with

with $L$ denoting the Lie derivative of the vector field . Such divergence can be written in local co-ordinates as,

Now for any scalar function we can also define a vector field on the manifold using inner products for all vectors $v_{\theta}$ at point $v$ on the tangent space ,

With these identities, the Laplace-Beltrami operator ($\Delta$) becomes,

The Langevin equation comprises two terms – a drift term and a diffusion term; where the latter is represented by the Laplace-Beltrami operator (diffusion with ‘constant’ infinitesimal variance with respect to the metric) when diffusion occurs on a Riemannian manifold. While the gradient has the form in Eqn. , using Ito’s calculus, one can show the non-linear mapping of the martingale $d\mathbf{b}(t)$ becomes,

$$d\tilde{\mathbf{b}}_{t}=\frac{1}{2}\frac{1}{\sqrt{\left|g\left(\theta\right)\right|}}\partial_{i}\left(\sqrt{\left|g\right|}g^{ij}\right)dt+\sqrt{g^{ij}}d\mathbf{b}_{t}$$

The probability density $p(\theta)$ also has to be altered to yield the correct invariant density on the manifold; this can be attained by the following transformation

$$\tilde{p}(\theta)=p(\theta)\frac{1}{\sqrt{\left|g(\theta)\right|}}$$

Combining all of the transformations we obtain the Langevin equation on the manifold,

Such flows are invariant under transformation of random variables and covariant under re-parameterisation. Specifically under the Bayesian framework, the metric becomes the expected Fisher information in combination with the negative Hessian of the log-prior [[3](#_ENREF_3)].

**S3.2 Deterministic approach** – sufficient statistics of the generative model form the co-ordinate basis

The evolution of the variational free-energy could be described on a Riemann manifold by augmenting the first order gradient flow using a Fisher information metric [[4](#_ENREF_4),[5](#_ENREF_5)]. On a Euclidean manifold, the minimization of variational free-energy involves

This simply says that the flow of parameters (e.g. means and co-variance of a Normal distribution; $\{\mu,\sigma,\ldots\}\in\theta$) will induce the largest change in free-energy under a unit change in parameters. Notice that the inner products are defined on a Euclidean manifold.

Classical results from information geometry (Cencov’s characterisation theorem) tell us that, for manifolds based on probability measures, a unique Riemannian metric exists – the Fisher information metric. In statistics, Fisher-information is used to measure the expected value of the observed information. Whilst the Fisher-information becomes the metric for curved probability spaces, the distance between two distributions is provided by the Kullback-Leibler (KL) divergence. It turns out that if the KL-divergence is viewed as a curve on a curved surface, the Fisher-information becomes its curvature:

Gradient descent on such a manifold then becomes the solution of , subject to $KL_{sym}(\theta,\theta+d\theta)<\varepsilon$; i.e., the direction of the highest decrease in the free-energy, for the smallest change in the KL divergence. The solution of this optimization problem yields Amari’s natural gradient that replaces the Euclidean gradient by its Riemannian counterpart . This derivative is invariant under re-parameterisation of the approximate probability distribution, thereby helping us to break symmetries on the variational free-energy manifold.

This formulation has two important consequences – (a) from classical results in statistics, pre-conditioning of the free-energy gradient by the Fisher-information tells us that the variance of the estimator is bounded from below by the Fisher-information (Cramér-Rao bound) and (b) under a Normal distribution approximation of the posterior distribution, precision-weighted prediction errors under a Euclidean manifold are replaced by asymptotic dispersion and precision-weighted prediction errors under a Riemannian manifold.

Such constructs are already instantiated in advanced Bayesian filtering schemes, such as the SPM code-base (available from http://www.fil.ion.ucl.ac.uk/spm/) using Fisher-scoring – the gradient of variational free-energy is pre-multiplied by the inverse Fisher information metric. Notice that the metric in Fisher-scoring is simply the variance of the score function, while our derivation of the metric includes not only the metric for the likelihood but also that of the prior (instantiated as the Hessian of the prior).

The question that we now ask is whether we can deduce an optimization scheme that enables us to traverse the free-energy landscape? In other words, find the geodesic to local minima in the sub-manifold. There are two routes one can take to increase the statistical efficiency of the implicit optimisation – first, we can formulate the Hessian operator on the Riemannian manifold in terms of the Laplace-Beltrami operator (Section S3.1) or we can retain a first-order approximation and formulate descent directions that are orthogonal to the previous descent directions. Such Krylov sub-spaces are well-known in numerical analysis with the *conjugate gradient-descent* algorithm providing one such example (Figure S1). Routinely used in optimization, conjugate gradient descent methods have been used for gradient descent on manifolds traced out by energy functions such as the variational free-energy [[6](#_ENREF_6),[7](#_ENREF_7)]. Simply such a scheme amounts to,

$$\begin{matrix} \theta_{i}=\theta_{i-1}+\alpha H_{i} \\ H_{i}=-G_{i}+\beta H_{i-1} \\ \beta=\frac{\nabla_{i}^{T}\nabla_{i}}{\nabla_{i-1}^{T}\nabla_{i-1}}\to^{Riemannize} \frac{\tilde{\nabla}_{i}^{T}\nabla_{i}}{\tilde{\nabla}_{i-1}^{T}\nabla_{i-1}} \end{matrix}$$

For $\beta$ we have used the Fletecher-Reeves instantiation on a curved manifold; other update rules such as Polak-Ribière, Hestenes-Stiefel or Dai-Yuan can be similarly lifted to a Riemannian manifold [[8](#_ENREF_8)]. All of these conjugate gradient descent formulas have a problem – one cannot add two vector fields $H_{i}$ and $H_{i-1}$ on a Riemannian manifold. This is because they exist on different tangent manifolds. $H_{i-1}$ should undergo parallel transport to the tangent manifold containing $H_{i}$ using a connection (a gauge) field. In our case, this is the Levi-Civita connection described in Section S2.

Parallel-transport requires the solution of a second-order differential equation. Analysis shows us that the natural gradient is simply the first order approximation of the parallel transport – that we pursue in terms of solving geodesic equations for the sufficient statistics. For the Laplace approximation, we could derive the Christoffel symbols analytically (Section S4), while for more complicated probability distributions we need to resort to a generic transport procedure (Figure S2). Namely, we use the Riemann exponential map for mapping the vector field on the tangent manifold to the geodesic described on the manifold , whereas a Riemann logarithmic map represents the transformations of vector-fields from the manifold to the tangent manifold ,

$$\begin{matrix} \exp_{\theta}(A)=\theta^{1/2}\exp(\theta^{-1/2}A\theta^{-1/2})\theta^{1/2} \\ \log_{\theta}(A)=\theta^{1/2}\log(\theta^{-1/2}A\theta^{-1/2})\theta^{1/2} \end{matrix}$$

The geodesic is first approximated using standard projection method [[9](#_ENREF_9)]. Then using the exponential and logarithm maps a Schild’s ladder [[10](#_ENREF_10)] is instantiated as following: Let $\theta_{I}$ and $\theta_{F}$ denote the initial and final points on the geodesic that the vector field is to be transported to. We start by calculating $n_{1}=exp_{\theta_{I}}(H)$ and the midpoint $m_{1}$ between the geodesic segment joining $n_{1}$ and $\theta_{1}$. We then trace out the geodesic from $\theta_{I}$ through $m_{1}$ for twice its length, tracing out a new point $n_{2}$. This scheme is repeated until we reach $\theta_{F}$. After the vector field $H$ has been parallel transported to $\theta_{F}$, we are in a position to use parameter updates as detailed in Eqn. . Numerical instantiation of this algorithm shall be presented elsewhere [[11](#_ENREF_11)].

1. Jordan R, Kinderlehrer D, Otto F (1999) The variational formulation of the fokker-planck equation. SIAM J Math Anal 29: 1-17.

2. Hsu E (2002) Stochastic Analysis on Manifolds: American Mathematical Society.

3. Sengupta B, Friston KJ, Penny WD (2015) Gradient-based MCMC samplers for dynamic causal modelling. Neuroimage.

4. Amari S (1995) Information geometry of the EM and EM algorithms for neural networks. Neural Networks 8: 1379-1408.

5. Tanaka T (2001) Information geometry of mean-field approximation. In: Opper M, Saad D, editors. Advanced Mean Field Methods: Theory and Practice: The MIT Press. pp. 259-273.

6. Honkela A, Raiko T, Kuusela M, Tornio M, Karhunen J (2010) Approximate Riemannian Conjugate Gradient Learning for Fixed-Form Variational Bayes. Journal of Machine Learning Research 11: 3235−3268.

7. Hensman J, Rattray M, Lawrence ND. Fast variational inference in the conjugate exponential family; 2012.

8. Nocedal J, Wright S (2006) Numerical Optimization: Springer.

9. Hairer E, Lubich C, Wanner G (2004) Geometric Numerical Integration: Structure Preserving Algorithms for Ordinary Differential Equations: Springer.

10. Misner C, Thorne K, Wheeler J (1973) Gravitation: W.H. Freeman.

11. Sengupta B, Penny WD, Friston KJ (2016) Information geometric variational learning. (under preparation).
