# Supplementary material for: Towards a Neuronal Gauge Theory
Source: PLoS Biol. 2016 Mar 8;14(3):e1002400. doi: 10.1371/journal.pbio.1002400 (PMC4783098; doi:10.1371/journal.pbio.1002400)
Supplement: S4 Text — (DOCX) [file pbio.1002400.s008.docx]

**S4 Text. The geometry of a univariate Normal distribution**

The normal distribution, a member of the exponential family, does not enjoy the geometry of a Euclidean manifold. The Laplace-approximation of a variational density (see [Friston, Mattout et al. 2007](#_ENREF_22)) rests on approximating the posterior distribution with a Normal distribution. Therefore, it becomes instructive to understand its geometry. Calculation shows that the Riemannian metric (the Fisher information) simply reads $g(\mu,\sigma)=diag\left[\sigma^{-2}\text{ }2\sigma^{-2}\right]$ for a univariate Normal. Further algebra shows that the non-zero terms of the Levi-Civita connection for a univariate Normal distribution are

$$\Gamma_{1,2}^{1}=-\frac{1}{\sigma},\Gamma_{1,1}^{2}=\frac{1}{2\sigma},\Gamma_{2,2}^{2}=-\frac{1}{\sigma}$$
