# Supplementary material for: Towards a Neuronal Gauge Theory
Source: PLoS Biol. 2016 Mar 8;14(3):e1002400. doi: 10.1371/journal.pbio.1002400 (PMC4783098; doi:10.1371/journal.pbio.1002400)
Supplement: S5 Text — (DOCX) [file pbio.1002400.s009.docx]

**S5 Text. Symmetry-aware algorithms**

A technically, prescient issue is how to use these notions to identify models via symmetry [[1](#_ENREF_1)]. The (pure) mathematical tool that allows us to do this is topological group theory [[2](#_ENREF_2),[3](#_ENREF_3)]. An important lead using group theory for statistical inference is the development of a novel class of Markov chains called *orbital Markov chains* [[4](#_ENREF_4)], that could potentially be used to draw samples from the equivalence class of a probability measure. In the absence of symmetries, orbital Markov chains are equivalent to standard Markov chains. Unsurprisingly, taking symmetries of the proposal distribution into account leads to a faster convergence (rapid mixing) of the Markov chain [[5](#_ENREF_5),[6](#_ENREF_6)]. Equivalence classes in such orbital Markov chains are deduced using a well-known product replacement algorithm [[7](#_ENREF_7)]. Such an algorithm allows generating an approximately uniformly distributed random element of a finite group by instantiating a random walk on the group. Under a variational inference framework, our differential geometry algorithm instantiates a similar symmetry-awareness (see Section S3). The challenge remains in understanding the symmetry groups for directed Bayesian networks via established tools of geometry and group theory, helping us forge a gauge theory that is not only useful for understanding biotic self-organization but also in the statistical analysis of data.

1. Nishihara R, Minka T, Tarlow D (2013) Detecting Parameter Symmetries in Probabilistic Models. arXiv preprint arXiv:1312.5386.

2. Diaconis P, Gupta SS (1988) Group representations in probability and statistics.

3. Eaton ML (1989) Group Invariance Applications in Statistics: Institute of Mathematical Statistics.

4. Niepert M. Markov chains on orbits of permutation groups; 2012.

5. Niepert M. Lifted probabilistic inference: An MCMC Perspective; 2012.

6. Shariff R, György A, Szepesvári C. Exploiting symmetries to construct efficient MCMC algorithms with an application to SLAM; 2015.

7. Pak I. The product replacement algorithm is polynomial; 2000. pp. 476-485.
